# Biodegradation of Dental Resin-Based Composite—A Potential Factor Affecting the Bonding Effect: A Narrative Review

**DOI:** 10.3390/biomedicines10092313

**Published:** 2022-09-18

**Authors:** Xinwei Guo, Yiyan Yu, Shang Gao, Zhimin Zhang, Hongyan Zhao

**Affiliations:** 1 School and Hospital of Stomatology, Jilin University, Changchun 130012, China; 2 The Fifth Affiliated Hospital of Sun Yat sen University, Zhuhai 519000, China

**Keywords:** biodegradation, resin, dentistry, composite, esterase

## Abstract

In recent years, although resin composite has played an important role in the restoration of tooth defects, it still has several disadvantages, including being biodegraded by saliva, bacteria and other enzymes in the oral cavity, which may result in repair failure. This factor is not conducive to the long-term survival of the prosthesis in the mouth. In this article, we review the causes, influencing factors and prevention methods of resin biodegradation. Biodegradation is mainly caused by esterase in saliva and bacteria, which breaks the ester bond in resin and causes the release of monomers. The mechanical properties of the prosthesis can then be affected. Meanwhile, cathepsin and MMPs are activated on the bonding surface, which may decompose the dentin collagen. In addition, neutrophils and residual water on the bonding surface can also aggravate biodegradation. Currently, the primary methods to prevent biodegradation involve adding antibacterial agents to resin, inhibiting the activity of MMPs and enhancing the crosslinking of collagen fibers. All of the above indicates that in the preparation and adhesion of resin materials, attention should be paid to the influence of biodegradation to improve the prosthesis’s service life in the complex environment of the oral cavity.

## 1. Introduction

A tooth defect is the loss of hard tissues of the tooth caused by caries, trauma and other factors, and can seriously affect the patient’s aesthetics and chewing function [1,2]. Therefore, the defect needs to be restored as soon as possible. In the past, the material commonly used to fill caries was amalgam, but the mercury content causes environmental pollution. In addition, the use of amalgam to fill cavities depends on mechanical retention rather than the use of adhesives, which means that many hard tissues will be removed to shape a perfect retention form [3,4,5]. To solve these problems, resin composites have been gradually replacing the role of amalgam in recent years [6]. Resin is widely used in dentistry nowadays because of its good fluidity, similar color to teeth, chemical bonding with dentin, and it causes less damage to hard tissues of the tooth. In addition to repairing caries, resin composites can also be combined with ceramics for full crowns and onlays. Hard ceramic restorations significantly differ in elastic modulus from dentin, which can sometimes lead to tooth fractures. Nevertheless, when combined with resin, the ductility and elastic modulus of the new restoration product are much closer to that of dentin, so the probability of tooth fracture can be reduced [7,8]. Widely applied Vita Enamic (Vita Zahnfabrik, Bad Säckingen, Germany) and Lava Ultimate (3M ESPE, Sao Paulo, MN, USA) are both resin–ceramic composites. To sum up, resin and its composite materials have occupied a vital position in prosthodontics. Common resin-based composite materials in dentistry include resins for filling, self-curing resins for temporary crowns, resin adhesives for restoration bonding, and the abovementioned resin–ceramic composites, etc., [9]. What is more, components making up resin mostly incorporate three parts. One is a polymer matrix formed by the polymerization of multiple monomers, which mainly determines the fluidity of the resin; another comprises inorganic fillers such as glass and quartz, which mainly determine the hardness of the resin; and the last is the silane coupling agent used to improve the interface bonding between the matrix and the inorganic fillers [10]. Additionally, the initiator system and polymerization inhibitor are also included in the resin matrix.

However, the resin also has some disadvantages. Compared with amalgam, resin-based materials have a higher failure rate and replacement frequency, and they are more prone to secondary caries [11,12], which may be related to incomplete polymerization and biodegradation of the resin. Resin composite, which is a high-molecular-weight polymer, is crosslinked with macromolecular monomers, including bisphenol a-diglycidyl methacrylate (Bis-GMA), triethylene glycol dimethylacrylate (TEGDMA) and urethane dimethylacrylate (UDMA). It can be polymerized under certain initiation conditions, such as chemical reaction initiation, photoinitiation and thermal initiation. In recent years, high-temperature and high-pressure initiation systems have been gradually applied [13]. However, initiation conditions cannot polymerize all monomers in the resin (especially those in deeper parts). There will still be a few monomers in a free state, thus leading to their release from the cured resin. Some studies demonstrate that the complete polymerization rate of monomers does not exceed 75% [10]. Additionally, there are a large number of unprotected ester bonds in these monomers that are unstable, and that can be easily hydrolyzed by esterases or other enzymes in the oral environment. Then, some by-products are released, such as 2,2-Bis[4(2,3-hydroxypropoxy)phenyl]propane (Bis-HPPP), triethylene glycol (TEG) and methacrylic acid (MA) [14]. These monomers can be detected by high-performance liquid chromatography (HPLC), UV spectrum and mass spectrometry [15]. This kind of degradation dominated by biological factors is called “biodegradation”. The by-product, Bis-HPPP, is the sign of biodegradation in the Bis-GMA-based resin matrix [16]. Esterase in the oral cavity can be derived from bacteria and saliva, which is a potential factor leading to repair failure, but this factor is often ignored by clinical practitioners. They mostly focus on studying how to improve the polymerization rate of resin monomers, ignoring the fact that large amounts of bacteria in the mouth can also destroy the chemical structure of resins. Biodegradation usually increases the surface roughness of the resin, affects the edge fitness, and augment the adhesion of bacteria. This results in the enlarging of the bacterial biofilm, and forms a vicious circle aggravating degradation [17]. In addition, most of the adhesives currently used in surface bonding are composed of resins. In this way, biodegradation is more likely to destroy the adhesive layer, which has a negative effect on the bonding effect of the prosthesis. Another form of biodegradation is the degradation of dentin collagen fibers mainly caused by matrix metalloproteinase (MMP), resulting in cracks between resins and dentin [18,19]. Under normal circumstances, the uncured resin can penetrate the dentin collagen fiber network to form a hybrid layer after the tooth surface is treated with primer. If this “hybrid layer” is attacked by esterases and MMPs at the same time, the resin layer and the collagen fiber layer will be damaged.

All in all, biodegradation is an essential factor affecting resin restoration. The internal environment of the oral cavity is complex, and the prosthesis exists in this environment of saliva and bacteria for a long time, experiencing variable thermal cycles. The bonding interface between the resin and teeth is prone to being affected. Once this interface is damaged, some problems may occur, such as secondary caries and the failure of restoration, etc. Therefore, paying attention and taking necessary measures to reduce biodegradation is crucial [20]. Thus, this article reviews the factors causing the biodegradation of resin-based materials, and summarizes measures to reduce biodegradation, to provide a reference for the rational use of resin-based materials in clinical practice.

## 2. Factors Causing Biodegradation

### 2.1. Esterases

#### 2.1.1. Esterase-Like Activities in Saliva

Saliva contains both inorganic components, such as calcium and magnesium, and organic components, such as protein [21]. Among the protein components, some esterases are related to the biodegradation of resin-based materials [22]. Human saliva-derived esterase (HSDE) is a salivary enzyme that exhibits vigorous degradation activity on Bis-GMA, one of the most common monomers in resin composites and adhesives [23]. Cholesterol esterase (CE)-like activity and pseudocholinesterase (PCE)-like activity can be detected in saliva [15]. Studies have shown that commercial CE and PCE can hydrolyze the ester bond in the resin matrix [23]. HSDE, which exhibits CE- and PCE-like activity, also has this effect [24].

Cholesterol esterase (CE) is produced by monocyte macrophages and exists in normal and inflammatory gingiva [25]. When the non-specific immune response of acute inflammation is initiated, or external prostheses have entered the oral cavity, the release of CE will increase [15]. PCE is a form of glycoprotein that widely exists in various tissues and has low specificity for acetylcholine. It can hydrolyze not only other choline esters [26], but also a part of the ester bonds in resin monomers [27]. These two kinds of esterases have a strong ability to degrade Bis-GMA, TEGDMA, HEMA and other monomers containing esters in resin composites [28]. The two esterases also have corresponding specific substrates, p-nitrophenylbutyrate (p-NPB) and butyryl thiocholine, respectively, which are used to detect the activity of CE and PCE [15]. Similar to other esterases, the activity of these two esterases can also be inhibited by the esterase inhibitor phenylmethylsulfonyl fluoride (PMSF), which means that biodegradation can be reduced to some extent [25]. Although HSDE in the oral cavity is generally manifested as CE-like and PCE-like activity, CE and PCE only represent a small fraction of saliva components. There are also other complex proteins taking part in the degradation process of resin [29]. Cui et al. [29] found that albumin in saliva is the main constituent in the biodegradation of resin through mass spectrometry, exhibiting CE-like and PCE-like activity, with p-NPB as the substrate. Furthermore, when albumin and zinc-alpha2-glycoprotein (ZAG) in saliva form a protein complex in the ratio of 5:1, the ability to degrade resin matrix is significantly improved. This may be because ZAG can improve the clearance of degradation products at the active site of albumin, and this process is more pronounced in alkaline environments.

In an in vitro test, after Bis-GMA-based resin was incubated in buffer containing CE for 8 days, the abundance of Bis-HPPP could be detected [15]. On the 30th day, microleakages could be observed at the bonding interface between the tooth and resin, and the penetration of bacteria rose [30]. In the vicinity of the microleakages, a large number of plaques gathered, causing or aggravating secondary caries. Studies have proved that human saliva can degrade some commercial resin composites, including Filtek Z250 (3M ESPE) and Spectrum TPH (L.D. Caulk). Both of these contain Bis-GMA, TEGDMA and polyurethane-modified Bis-GMA. However, polyurethane-modified Bis-GMA is more stable and resistant to hydrolysis [31].

The presence of esterase in saliva has an adverse impact on the mechanical properties of the resin. After aging for 180 days in an environment containing artificial saliva and esterase, the radial tensile strength and elasticity of resin composite dramatically declined [32], and the composite also showed the lowest fracture toughness [23]. On the 12th day alone, the hardness of the thermally polymerized acrylic resin in saliva decreased, and the roughness began to increase on the 24th day [33]. At the same time, the shear strength and microtensile strength of the resin soaked in saliva dropped sharply in a very short time [34,35]. Furthermore, biodegradation also softens the surface layer of the resin, leading to mechanical wear during chewing. Once the softened surface layer is removed, the newly exposed layer is continually eroded, resulting in the breakage of the interface structure [10]. The mechanical characteristic of restoration is an important criterion for evaluating its durability. The cold and heat circulation in the mouth and the complex enzymes in the saliva will reduce the mechanical strength of the resin, which is also the reason that resin materials are not as durable as metal materials [11].

#### 2.1.2. Esterases from Bacteria

As one of the cavities in the human body that is in contact with the outside world, the oral cavity contains many bacteria. These bacteria usually exist on the teeth, tongue, mucosa and restoration surface in the form of biofilm. Among these bacteria, *Streptococcus mutans (S. mutans)* is the chief culprit of dental caries; therefore, it has been widely researched [36]. *Streptococcus mutans* can ferment sucrose to produce acid, then demineralize hard tissues and cause caries. In recent years, *S. mutans* has been found to be the main bacteria species causing biodegradation [37,38]. When the Bis-GMA-based resin was co-cultured with *S. mutans*, it was found that *S. mutans* considerably enhanced the surface roughness of the resin composite [38], and Bis-HPPP could be detected after 14 days [37]. In addition, bacterial esterase can also increase the shrinkage of methacrylate resin and reduce the mechanical properties of the material, such as microhardness, bending strength, radial tensile strength and elastic modulus [39,40]. When the surface of the resin becomes rough, the adhesion of bacteria increases. Then, the esterase that the bacteria produce will aggravate biodegradation, and the increasing acid production will also accelerate the demineralization of hard tissue. Moreover, when the mechanical properties of the resin are weakened, restorations tend to fall off. In conclusion, *S. mutans* can weaken the adhesion. Up to now, researchers have conducted more in-depth studies on bacterial esterase.

Basic Local Alignment Search Tool (BLAST) comparison shows that there are five possible esterase-related genes in *S. mutans*, named *SMU-118c*, *SMU-400, SMU-643, SMU-1443c* and *SMU-1678*. However, when testing the hydrolysis ability of proteins expressed by these genes, it was found that only one esterase, *SMU-118c*, acted as a true hydrolase for nitrobenzene substrates [41]. Huang et al. [42] cocultured the wild strain and *SMU-118c* gene knockout strain of *S. mutans*, UA159, with resin composite, respectively, for 30 days. They showed that compared with the wild strain, Bis-HPPP release in the *SMU-118c* gene knockout group decreased. However, when *SMU-118c* was supplemented, the release of Bis-HPPP recovered, indicating that esterase *SMU-118c* can indeed hydrolyze Bis-GMA. In fact, *S. mutans* UA159 has the most robust esterase activity among all strains of *S. mutans* with p-NPB as the substrate. This activity has been proved to be related to the degradation of resin composites [31].

*SMU-118c*, a 280-base-pair gene located in *S. mutans* UA159, plays a major role in biodegradation. It can maintain its biological activity in acidic and neutral environments for 21 days, which means that under cariogenic conditions, it can hydrolyze resin for a relatively long time [41,43]. In neutral (pH = 7) and cariogenic (pH = 5.5) environments, Bis-GMA and TEGDMA can be degraded into Bis-HPPP and TEG [41]. However, the release amount of Bis-HPPP is different in acid and neutral environments. Borge et al. [44] evidenced that the release of Bis-HPPP in an acidic environment is more than that in a neutral environment. This study shows that biodegradation is more likely to occur under cariogenic conditions. In other words, the biological environment of secondary caries can aggravate biodegradation.

The oral cavity is a multibacterial environment. Although *S. mutans* is the main bacteria causing biodegradation, there are also other bacteria in the oral cavity that can degrade resin-based materials. *Enterococcus faecalis*, a common Gram-positive bacterium, often exists in infected root canals. It is usually considered to be related to periapical infection [45]. Marashdeh et al. [27] cultivated a certain concentration of *Enterococcus faecalis* with resin composite for 30 days. Then, the release of Bis-HPPP could be detected. They found that *Enterococcus faecalis* exhibited esterase-like degradation activity on dental methacrylic resin, which could accelerate the degradation of the bonding interface, increase the proliferation of bacterial biofilm, and further their penetration into the root canal system. Interestingly, in addition to esterase activity, *Enterococcus faecalis* also showed MMP activity. This finding will be described in detail later in this review.

In addition to *S. mutans* and *Enterococcus faecalis*, studies have shown that *Streptococcus sanguis* and *Streptococcus Gordon* can also reduce the bending strength of resin and change the surface morphology [46]. However, whether this process is related to biodegradation needs further study.

The by-products of resin degradation may also further enhance the virulence of bacteria. Studies have proven that by-products such as TEG and Bis-HPPP can accelerate the reproduction of *S. mutans* and boost the expression of related virulence genes [47,48]. Bis-HPPP can upregulate the virulence factors *gtfB/C*, *gbpB*, *comC/D/E* and *atpH* and the glucosyltransferase activity in *S. mutans*, especially under cariogenic acid conditions (pH = 5.5) [49]. Furthermore, TEG can upregulate the expression of *gtfB/C*, *GbpB*, *comC*, *comD* and other genes related to acid production, and increase the abundance of proteins related to biofilm formation and acid tolerance [17]. It can be seen that these by-products elevate the cariogenic ability of *S. mutans*. Nevertheless, some studies have shown that the monomers HEMA, EGDMA and DEGDMA do not promote the growth of *S. mutans* [50]. As a result, whether monomers can enhance bacterial virulence depends on their concentration and type.

#### 2.1.3. Esterase from Neutrophils in Gingival Crevicular Fluid

Neutrophils, as notable components of the immune system, are rich in azure granules with strong proteolysis. In addition to phagocytizing, killing and dissolving bacteria and fungi [51], neutrophils can also cause tissue damage in the inflammation process [52]. What is more, they can also exhibit esterase-like activities. Neutrophils mainly exist in the gingival crevicular fluid in the oral environment, and can directly contact the edge of the prosthesis. The esterase activity of neutrophils can directly degrade the subgingival adhesive containing resin components and the bonding interface. Gitalis et al. [53] found that the esterase-like activity of neutrophils promoted the release of Bis-HPPP in Bis-GMA and accelerated the degradation of the photocurable resin. Neutrophil esterase is similar to HSDE, which can hydrolyze the ester bond in Bis-GMA. However, its degradation rate at the tooth–resin interface is much faster than that of HSDE, meaning that there are also other enzymes in neutrophils mediating biodegradation, such as cathepsin G and myeloperoxidase [16]. Cathepsin G is a serine protease that can hydrolyze dentin collagen fibers [54]. This process can also lead to interface damage and then provide a path for bacterial invasion, causing secondary caries. Neutrophils also release myeloperoxidase and hypochlorite. The former can break the vinyl ether bond; the latter can catalyze the hydrolysis of the ester bond in resin [16]. Some ether bonds in Bis-HPPP react with water, forming vinyl ether bonds, which are then cleaved by myeloperoxidase [16,55].

### 2.2. Protease

Biodegradation occurs not only in the resin component but also in the dentin component. Different from enamel, the content of organic matter in dentin is higher, reaching 30%. These organic components are mainly composed of collagen fibers. They can be decomposed by protease secreted by bacteria and cells. The final result is dentin demineralization, which expedites the process of dentin caries [56].

#### 2.2.1. Matrix Metalloproteinase

MMPs are proteolytic enzymes that depend on zinc and calcium ions. MMPs that degrade collagen in oral biodegradation mainly originate from the endogenous dentin matrix and exogenous bacteria.

Endogenous MMPs inherent in dentin are mainly secreted by odontoblast cells during dentin formation and mineralization, including MMP-1, 2, 3, 8 and 9. They can decompose the water-rich and loose dentin collagen fibers, mostly Collagen I, in the hybrid layer formed by adhesive and dentin [54]. The incomplete penetration of resin monomers into the collagen network makes it particularly susceptible to degradation by MMPs [57]. Typically, MMPs are “locked” within hard tissues during mineralization, but some chemical and physical conditions can activate them [58]. Especially under acid cariogenic conditions, endogenous MMPs can be activated through a series of processes. When the *S. mutans* concentration in the oral cavity increases, the esterase from the bacteria will degrade the resin matrix in the adhesive. At the same time, the resin in the hybrid layer can also be washed out by residual water, resulting in cracks between the dentin and adhesive. Meanwhile, MMPs are activated by the acid produced by bacteria and the acid resin monomer in the adhesive, and consequently, the exposed dentin collagen fibers in the hybrid layer are hydrolyzed [59,60,61]. In addition to the cariogenic acid environment, Mazzoni et al. [62] demonstrated that both total etching and self-etching can activate MMP-2 and MMP-9 related to hydrolysis of the dentin collagen fibers. What is more, MMPs can also be activated by other proteases, sulfhydryl modifiers and reactive oxygen species, as well as physical conditions (low pH, heat, etc.) [63,64]. It is suggested that there is a synergistic relationship between MMPs and cathepsins (CTs). The acidic activation of CTs may further activate MMPs [65]. However, compared with the degradation rate of resin caused by esterase, the rate of hydrolysis of dentin collagen fibers by MMPs is considerably slower [66]. Therefore, resin protrusions are first destroyed in the hybrid layer, and then the collagen fiber network is slowly eroded.

In addition to endogenous MMPs, some bacteria also release MMPs in biofilm to degrade collagen fibers. Heterotrophic aerobic bacteria are usually the main microorganisms secreting MMPs, because they rely on external proteins as their nutrients. Studies have revealed that *Enterococcus faecalis* exhibits MMP-8- and MMP-9-like activities, and thus can hydrolyze the dentin collagen on the bonding interface. This process allows bacteria to invade the root canal system [67]. Incubation of *Enterococcus faecalis* within demineralized dentin for 24 h can lead to the degradation of dentin collagen [67]. Furthermore, *Pseudomonas*, *Vibrio*, *Bacillus*, *Proteus* and *Serratia* can secrete MMPs, although most of the MMPs they produce belong to pathogenic virulence factors [68].

#### 2.2.2. Cysteine Cathepsin

As another enzyme that can hydrolyze dentin collagen, cysteine cathepsin often exists in normal and carious dentin together with MMPs [69]. Tersariol et al. [70] first illustrated that cysteine cathepsin exists in odontoblast and dental pulp tissue in 2010. Among CTs, CT-K accounts for 98% of the anti-collagen activity. It can hydrolyze the regions outside the helical fibers, as can other CTs, and cut the helical collagen into many fragments at multiple sites [71,72]. CTs can also be treated as participants in biodegradation.

### 2.3. Organic Acid Degradation

Apart from esterase and MMPs, the continuous metabolism of dental plaque can produce accumulated lactic acid. Lactic acid can not only demineralize hard tissue, but also promote the degradation of resin. This is because the polar functional groups in lactic acid can attract a lot of hydrogen bonds and improve the water absorption of resin materials. The resin’s crosslinking structure expands, and the residual monomers are easy to move out [73,74]. However, this form of degradation only accounts for a small proportion of biodegradation.

## 3. Process of Biodegradation

### 3.1. Biodegradation Effect on Resin Restoration

The restorative resin composite is usually directly exposed to the salivary environment, and thus will be affected by intraoral esterases [28]. As mentioned above, saliva and bacteria in the mouth contain a number of esterases, which can hydrolyze the ester bond in resin, contributing to the release of monomers. Resin is polymerized by a variety of monomers, so once the monomers are released excessively, it will age and its mechanical properties will weaken to the extent that the resin composite cannot withstand the normal occlusal force. The final result is that it falls off and is damaged [34,35].

### 3.2. Biodegradation Effect on Resin–Tooth Bonding Interface

If the bonding interface is supragingival, the biological environment where the tooth contacts the prosthesis is mostly in the saliva. The targets of biodegradation include resin-based restorations, adhesives and dentin collagen fibers [37,54]. Firstly, the degradation rate of esterase from saliva and cariogenic bacteria is fast, making the resin close to the bonding interface degrade partially and produce microcracks. At the same time, the surface roughness of the resin increases, and more cariogenic bacteria colonize, thus accelerating the degradation of resin [33,38]. The main component of the adhesive is methacrylate, which will be attacked by esterase. In this way, the adhesive layer is slowly damaged, bringing about more bacterial invasion. A large number of cariogenic bacteria produce acid, making the microenvironment of the bonding surface more conducive to the metabolism and reproduction of bacteria. As a result, the demineralization of hard tissues is exacerbated, forming a positive feedback cycle and leading to more serious secondary caries and biodegradation [28,54]. Simultaneously, residue water in the hybrid layer also influences biodegradation. Most methacrylate adhesives are hydrophobic. If the adhesive is applied to aqueous dentin, the nanophase will be separated [75]. In other words, the hydrophobic adhesive will not thoroughly infiltrate the collagen fiber net, resulting in cracks between the adhesive and dentin. All these processes contribute to more microleakages, and then more saliva and bacteria seep into the cracks, exacerbating the biodegradation process [59]. As a result, the dentin collagen fiber net is exposed and further demineralized under the bacterial acid production. In the acid environment caused by etching agents and bacterial acid production, MMPs in the dentin are activated and degrade collagen fibers, contributing to more profound destruction to the bonding interface [54,59,60].

If the bonding interface is subgingival, the factors involved in biodegradation are not only bacteria and saliva, but also neutrophils in the gingival crevicular fluid. The esterase secreted by neutrophils will accelerate the degradation of the resin, and the cathepsin secreted by them will directly attack the collagen reticulum [51,52,53]. At the same time, the released monomers can irritate the gingiva, aggravating the inflammatory reaction in the gingival sulcus, resulting in the release of more hydrolases.

**Figure 1:**
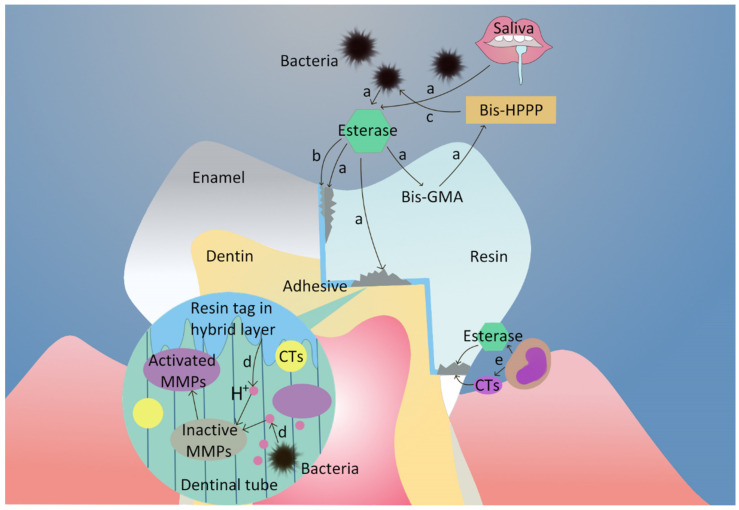
Process of biodegradation. (a) Esterase from cariogenic bacteria and saliva hydrolyzes Bis-GMA in resin restorations to produce Bis-HPPP. (b) Esterase destroys the adhesive layer. (c) Monomers in turn promote bacterial growth. (d) Acids from bacteria and etchants activate MMPs. Activated MMPs and CTs degrade dentin collagen fibers together. (e) Neutrophils in gingival crevicular fluid secrete esterase and CTs, then degrade Bis-GMA and dentin collagen fibers on the bonding surface.

It should be noted that monomers such as TEG, Bis-HPPP and UDMA can, in turn, promote the metabolism, proliferation and acid production of cariogenic bacteria. In such an environment, the biodegradation of the bonding surface will be further exacerbated [17,49,76]. Figure 1 demonstrates the process of biodegradation.

## 4. Monomers Released during Biodegradation and Their Biological Toxicity

### 4.1. Release of Monomers

The release of monomers mainly includes short-term release and long-term release [77]. The former means that monomers in the materials are released due to the limited curing conditions within the first 24 h [78,79]. The latter refers to the release of monomers in resin due to abrasion, saliva scouring, and enzyme degradation in saliva and bacteria during use.

There are two monomers related to biodegradation, macromers and small-molecule monomers. Macromers include TEGDMA, Bis-GMA, UDMA, etc. The former two are the main components of the resin matrix at present. After the surface layer of resin is degraded by esterase from saliva and bacteria, the macromers that are not fully polymerized in the deep layer will be slowly released. Small-molecule monomers include MA, TEG, Bis-HPPP, etc. They are derived from the polymerized resin matrix and the macromers whose weak ester bonds are destroyed by esterase [80]. For example, the ester bond of Bis-GMA is destroyed, and the by-products are Bis-HPPP and MA. In comparison, the ester bond breaking of TEGDMA is not as complete as that of Bis-GMA. Although TEGDMA contains two ester bonds, only one ester bond is broken, firstly, under the action of esterase. Most of TEGDMAs are only degraded into TEGMA and MA, and merely a small part of TEGMA breaks into TEG and MA, secondarily [14]. When the esterase *SMU-118c* of *S. mutans* was applied to Bis-GMA and TEGDMA, after 125 h, *SMU-118c* almost completely hydrolyzed Bis-GMA and produced a corresponding amount of degradation product, Bis-HPPP, within this period. In contrast, after 125 h, only 17.6 ± 4.4% and 11.9 ± 4.7% of TEGDMA monomers were hydrolyzed in acidic and neutral environments, respectively [41]. By comparison, there are few studies on the biodegradation of UDMA. Some studies show that UDMA is more stable than Bis-GMA and TEGDMA, with less monomer release [81,82]. Therefore, it is speculated that UDMA is more difficult to degrade. If degradation occurs, urethane and MA may eventually be generated.

### 4.2. Biosafety of Resin Monomers and Their Degradation Products

Bis-GMA is the main component of the resin matrix, and has a high release rate. Bis-GMA, a BPA derivative, also has toxic effects on cells [83]. Studies have evidenced that Bis-GMA blocks the cell cycle of exposed dental pulp cells in G2/M phase and promotes their apoptosis [84]. At the same time, it also induces the production of prostaglandins and reactive oxygen species, leading to dental pulp inflammation or necrosis [85]. Furthermore, Bis-GMA can damage the DNA of macrophages to some extent. It promotes DNA cleavage in macrophages by activating Caspase-3, Caspase-8 and Caspase-9 [86,87]. Moreover, it can also stimulate macrophages by inducing the expression of nitric oxide, inflammatory factors and surface antigens, thereby causing inflammatory responses [88,89,90]. If Bis-GMA enters the digestive tract, it will similarly impact the reproductive system and endocrine function [77].

TEGDMA and UDMA, monomers released from the resin, also have specific cytotoxicity. One effect of TEGDMA and UDMA on cells is that they can reduce the level of glutathione (GSH), a natural free radical scavenger that protects cells from ROS damage, and thus accelerate cell apoptosis [91,92]. Similar to Bis-GMA, they can also activate Caspase-3, 8 and 9, causing apoptosis in dental pulp cells [93,94]. Meanwhile, UDMA changes the normal morphology of dental pulp cells, lengthening or shortening normal spindle cells and reducing their activity [95]. Moreover, oxidative stress of gingival fibroblasts and pre-odontoblasts is initiated by TEGDMA through mitochondrial dysfunction [96,97]. The above are examples of the toxic effects of monomers on cells. From a macro perspective, these monomers can gradually infiltrate into the dental pulp cavity, periapical tissues and mucosa, resulting in pulpitis, periapical periodontitis, gingivitis and mucositis.

**Figure 2:**
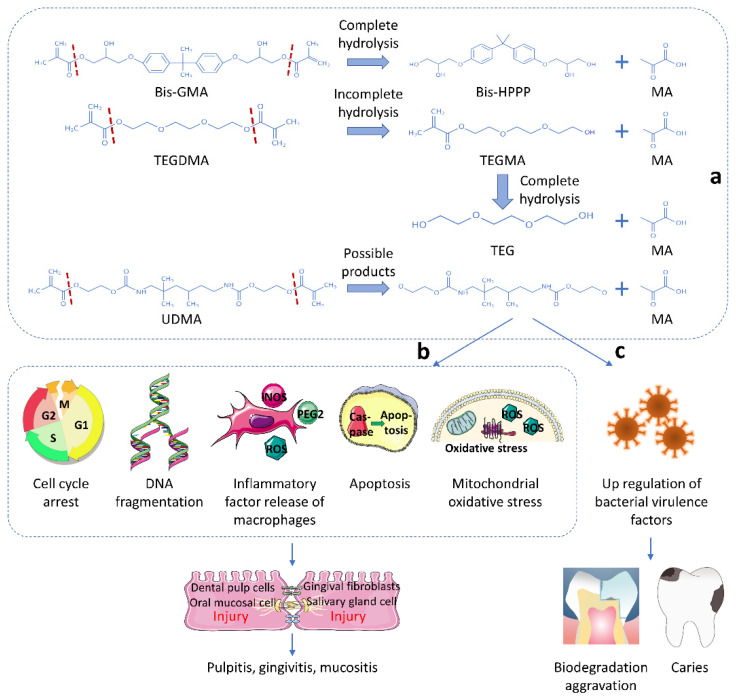
Biodegradation products of macromolecular monomers and the impact of monomers on oral health. (**a**) The ester bond of a macromolecular monomer is broken to form small monomers. (**b**) Monomers affect the physiological activities of cells, leading to local oral inflammation. (**c**) Monomers exacerbate the expression of bacterial virulence factors, and aggravate secondary caries and biodegradation.

Small monomers also exhibit the adverse effects mentioned above. Notably, Bis-HPPP, TEGMA and TEG can equally upregulate the virulence factors of *S. mutans*, aggravate their biodegradation ability on resin-based materials, and further the development of secondary caries [42,48,98]. Figure 2 describes the chemical structure of monomers, and shows their harmful influence on the oral environment.

## 5. Factors Affecting Biodegradation during Bonding

### 5.1. Resin Matrix

The resin matrix is mainly composed of Bis-GMA, TEGDMA and UDMA. Bis-GMA and UDMA are the most commonly used types, but their high viscosity limits the addition of inorganic fillers, and makes it difficult to obtain the required reinforcement effect. Therefore, it is necessary to add diluted organic monomers with low viscosity, such as TEGDMA, to reduce the viscosity of the system so that inorganic fillers can be added. The hydrolysis rate of Bis-GMA by esterase is fast, and the release of Bis-HPPP can be detected early in the oral cavity [41,99]. It is also reported that polyurethane-modified Bis-GMA is more stable and less biodegradable than unmodified Bis-GMA. Although the degradation efficiency of TEGDMA is not as high as that of Bis-GMA, it is more mobile and hydrophilic, and more easily penetrates the mouth, thus resulting in allergies [100]. UDMA-based resins containing more double bonds are prone to crosslinking reactions, and have a higher degree of polymerization and minor monomer release [7]. In order to further improve the polymerization reaction, currently, the method of high-temperature and high-pressure curing has been invented to take the place of light curing. This method will be introduced in detail later.

It is worth noting that the content of ester bonds in resins can directly affect the degree of biodegradation in the oral cavity. The biodegradation degree of resin with a high ester bond content is greater than that of resin with a low ester bond content [101].

### 5.2. Fillers

Fillers play a role in determining the hardness of the resin. Silane coupling agents are often used to assist the combination between fillers and resin matrix. Dental resin composites with higher silylated filler content are less susceptible to biodegradation. Moreover, non-silylated filler particles are more susceptible to degradation because of the lack of chemical bonding between fillers and matrix [102]. Research has shown that the increase in non-silylated silica filler particle content leads to the enhanced release of Bis-HPPP [103].

### 5.3. Adhesives

Currently, the most commonly used adhesives in clinical practice are resin-based, which are generally double curing. The main component of adhesive is methacrylate, which has a similar composition to resin composite. Therefore, adhesives are also more likely to be affected by saliva or bacterial esterase and undergo biodegradation [23]. Researchers have proved that adhesives produce Bis-HPPP after hydrolyzation by esterase [37]. When the adhesive is degraded, the close connection between dentin and the restorative resin composite will be destroyed, and microleakage will occur. As a result, restoration failure or secondary caries will take place.

Adhesives consist of both hydrophilic and hydrophobic parts; the two parts tend to separate in the aqueous hybrid layer [75]. The hydrophilic part passes through the interior of the hybrid layer to make contact with the aqueous dentin collagen fiber, while the hydrophobic monomer remains at the surface, which causes incomplete polymerization of the adhesive [58,104]. When the hydrophilic part of the adhesive moves towards the dentin collagen fiber net, it will form some microchannels in the hybrid layer, which become the routes for bacteria and saliva invasion, giving rise to further biodegradation. Therefore, the original intention of dentin wet bonding is good: water can keep the dentin collagen net from collapsing and make the adhesive penetrate the collagen net. However, too much water will affect polymerization. Interestingly, Pashley et al. [105] used ethanol instead of water to achieve better penetration of resin monomers in the collagen network and avoid the separation of adhesives.

### 5.4. Type of Etching

Phosphoric acid is used in the total etching system to remove the smear layer altogether. Then, the adhesive penetrates the exposed dentin collagen network to form a hybrid layer. On the contrary, the self-etching system does not entirely remove the smear layer, but partially dissolves it with an organic acid to demineralize the dentin at the lower part of the smear layer. Subsequently, the adhesive infiltrates into the collagen mesh below the smear layer to form a thinner hybrid layer [106].

Theoretically, all the excess water in the dentin tubules can become volatilized by the volatile solvent in the primer, so that the resin monomer can completely penetrate the collagen fiber network. However, due to the existence of residual solvents and the fluidity of the dentin fluid in the dentin tubules, the excess water cannot wholly volatilize, resulting in adhesives not forming a complete bond with the network [107].

The effects of the total etching and self-etching on biodegradation are different. Bourbia et al. [37] cocultured total-etched and self-etched specimens with *S. mutans* UA159, respectively, for 30 days, finding that more Bis-HPPP was released from the self-etched specimens. This is because the hydrophilicity of the self-etching adhesive can increase the water absorption on the bonding surface, which will make the ester bond of the internal resin monomer easier to be hydrolyzed by esterase [108]. Moreover, more residual water in the hybrid layer may also lead to a lower degree of monomer polymerization, thereby promoting biodegradation [54]. In addition, the fracture interfaces of total-etched and self-etched specimens treated by esterase are different. Serkies et al. [109] found that after immersing total-etched and self-etched specimens in esterase solution for 180 days, the fracture interfaces of the total-etched specimens migrated from the adhesive layer or resin layer to the hybrid layer, while the migration direction of the fracture interface in self-etched specimens was opposite [109]. This may be because there are more nanocavities in the self-etched bonding interface, which are formed due to the fact that resin monomers cannot completely penetrate the demineralized dentin fiber net. These cavities are the weak points of the early bonding interface [110]. Furthermore, since the self-etching system does not entirely remove the smear layer, the adhesive force between the smear plug and the resin is weak. In this condition, it may cause the complete or partial removal of the smear plug when the interface breaks, bringing out the opening of dentinal tubules and the secondary degradation of collagen fibers [109]. Moreover, the pH value of the adhesive in the self-etching system is lower, which may accelerate the decomposition of collagen [62]. All the above examples show that the self-etching system is more prone to initiating biodegradation than the total etching system.

Other studies have shown that the biomass of bacteria in the bonding interface of total etching and self-etching is also different. Huang et al. [54] indicated that there are more *S. mutans* in the self-etching treatment group than those in the total etching group. From the above we know that there are more nanocavities in the self-etching bonding interface mixing layer, which is conducive to the colonization of *S. mutans.*

## 6. Ways to Reduce Biodegradation

### 6.1. Protection of Ester Bonds by Fluorination

As most of the monomers in the resin have unprotected ester bonds, they are easily hydrolyzed by esterase-like activities in the oral environment [42]. Therefore, some researchers have proposed that fluorination of ester bonds in the monomers could improve their stability to a certain extent. The main idea is to add fluorinated groups to the hydrocarbon polymer substrate using chemical modification methods [111]. Another idea is to introduce homopolymerization or copolymerization involving fluorine-containing monomers. That is, replacing Bis-GMA or TEGDMA with fluorinated monomers to reduce the release of unreacted monomers, thus inhibiting biodegradation [112]. Delaviz et al. [113] showed that the synthesized fluorinated monomer was more resistant to the biodegradation of CE than Bis-GMA monomer. This is mainly because the hydrophobicity of fluorinated monomers can reduce the sensitivity of ester bonds to hydrolysis. In addition, the internal fluorocarbon chain of the modified monomer is larger than that of hydrocarbons, which provides steric hindrance to further protect ester bonds [113].

Although the fluorination of monomers in the resin can significantly alleviate biodegradation, the hardness, bending resistance and other mechanical properties of modified resins are also reduced; thus, it is not widely used in clinical practice [114]. Therefore, other methods for lowering ester bonds have also been studied. For example, Gonzalez-Bonet et al. prepared monomers based on ether bonds and found good anti-biodegradation ability [115].

### 6.2. Addition of Antibacterial Ingredients

#### 6.2.1. Antibacterial Fillers

Since esterase generated by bacteria in the oral cavity is one of the dominant causes of biodegradation, some antibacterial components can be added to the resin matrix as fillers, diminishing the presence of bacteria attached to restorations. These fillers include chemical drugs (chlorhexidine, fluoride, etc.) and nanomaterials (silver, copper, titanium dioxide, zinc oxide, etc.). They can be slowly released in the process of chewing wear [116]. However, their long-term release rate is poor, which means that they cannot maintain continuous antibacterial properties [117]. Consequently, in order to prolong the release of antimicrobial fillers, some researchers have linked them with mesoporous silica nanoparticles (MSNs). The flexible mesoporous structure and high surface area of MSNs make it possible to carry more drugs than traditional drug carriers, and the release rate is relatively stable. Therefore, MSNs have become one of the notable drug-transporting systems [118,119]. Lately, resins containing MSNs/Zn^2+^ complexes have been developed, and the antibacterial property of the synthesized new composite has equally been improved. When the addition of MSNs/Zn^2+^ accounts for 15% of the resin, the antibacterial rate of the composite even reaches 100%. Additionally, the mechanical properties of the synthetic resin are also significantly improved [120].

#### 6.2.2. Antibacterial Monomers

It can be seen from the above examples that most of the antibacterial components in the resin cannot be released at a remarkably stable rate. Moreover, chewing wear will gradually decrease the number of fillers. Hence, researchers have tried synthesizing antibacterial monomers that can polymerize a stable resin matrix. Compared with resins containing antibacterial fillers, the new materials show a property that once bacteria contact the surface of the material, they will be killed. Therefore, these materials are more stable and it is not easy to induce drug-resistant strains. The most commonly used antibacterial monomer is quaternary ammonium compounds (QAC), synthesized with ammonium salt molecule and methacrylic acid monomers [121]. These monomers contain positive charges, which can attract negatively charged bacteria. Thus, cell membrane permeability is changed, and the target bacteria dissolve and die [122]. At present, these naturally used QAC monomers include dimethyl hexadecyl methacryloxyethyl iodide (DHMAI), dimethylaminohexadecyl methacrylate (DMAHDM), 12-methacryloyloxy dodecylpyridinium bromide (MDPB), methacryloyloxyethyl hexadecyl ammonium chloride (DMAE-CB) and so forth [121,123,124]. They have a stable structure and remarkable antibacterial effect. In addition, QAC can also be combined with other nanoparticles to enhance the antibacterial effect. For example, in the material crosslinked by QAC and silver nanoparticles (AgNPs), bacteria can be killed when they contact the surface of quaternary ammonium resin. At the same time, AgNPs can penetrate the dentin tubules to kill bacteria [125]. In addition to crosslinking with nanoparticles, the combination of QAC and polyethyleneimine (PEI) can simultaneously play an antibacterial, -fungal and -viral role [126].

However, although QAC has an excellent antibacterial effect, it still has some limitations. Some studies have shown that introducing QAC into resin will increase the water absorption of resin and lead to a reduction in mechanical strength [127].

#### 6.2.3. Antibacterial Adhesives

Adding antibacterial agents to adhesives can reduce the accumulation of bacteria near the bonding interface, thereby reducing biodegradation. Stewart et al. [128] synthesized a form of resin-based adhesive containing 10 wt% otinidine hydrochloride/double hydrochloride silica co-assembled particles, and found that the otinidine released by this adhesive can inhibit the activities of bacterial esterase and collagenase. Adding ciprofloxacin to the adhesive can achieve a similar effect [22]. What is more, some nanoparticles, such as nanoparticles of silver or amorphous calcium phosphate, can be added to the adhesive. These nanoparticles penetrate the demineralized dentin tubules, kill the bacteria in the tubules and promote remineralization, thus enhancing the adhesion between the resin and tooth tissue [129].

### 6.3. Improvement of the Biological Stability of the Bonding Interface

#### 6.3.1. Inhibition of Protease Hydrolysis of Collagen

Since the hydrolysis of dentin collagen by tooth-derived MMPs and cysteine cathepsin is one of the principal reasons for the damage to the bonding interface, in recent years, inhibiting their activities has become a method to improve the stability of the bonding interface [109,130]. The most common MMPs and cysteine cathepsin inhibitors include Galardin and chlorhexidine [19,130]. The primary mechanism of Galardin is that there is a collagen-like chain inside it, which can combine with the active group of MMPs. Additionally, its hydroxamate structure (R-CO-NH-OH) competitively chelates with zinc ions in the catalytic region of MMPs [19], meaning that the MMPs cannot work efficiently.

In addition, some researchers have found that quaternary ammonium salts may act on the catalytic sites of MMPs through static electricity, thereby inhibiting the activities of MMPs [121]. Umer et al. [131] treated demineralized dentin with quaternary ammonium silane and found that the C-terminal crosslinked peptide (CTX) and C-terminal peptide (CTP) released from the degradation of dentin collagen by MMPs and cathepsin K decreased to a certain extent, thus proving the inhibitory activity of quaternary ammonium silane.

#### 6.3.2. Stabilization of Dentin Collagen Net

The use of a crosslinking agent can stabilize the structure of collagen, making it more resistant to enzymatic degradation. Common crosslinking agents are divided into three categories: chemical crosslinking agents, physical crosslinking agents and natural crosslinking agents. The most studied chemical crosslinkers include glutaraldehyde and carbodiimide (EDC). Glutaraldehyde has a great affinity for the amino group of collagens, and can increase the strength of collagen [132]. EDA can activate the free carboxyl groups of glutamic acid and aspartic acid in protein molecules, and form a stable covalent amide bond between proteins [133]. However, these two chemical reagents have certain cytotoxicity. Physical crosslinkers include ultraviolet activation (UVA) of riboflavin. This method mainly produces oxygen free radicals, mediating collagen crosslinking [134,135]. Natural crosslinking agents include proanthocyanidins (PAC) and genipin. Some studies have shown that adding PAC to resin adhesive can enhance the anti-degradation ability of dentin and maintain the stability of the bonding interface [136,137].

#### 6.3.3. Improvement of Resin Stability through High-Temperature and High-Pressure Initiation Conditions

At present, most resin composites used in clinics contain a photoinitiator system. Under light of 400–500 nm wavelength, the C=C bond in the resin monomers is disconnected, then, a C-C bond between adjacent monomers is formed during polymerization. In this process, the resin monomers that do not form a C-C bond are released and hydrolyzed by esterase in the oral environment, generating the by-product. Accordingly, it is very important to improve the polymerization rate and increase the stability of the resin. Studies have shown that the polymerization of resin monomers under high-temperature and high-pressure conditions can significantly reduce the release of unreacted monomers [138] compared with the photoinitiator system. In such conditions, the polymerization of resin will be sufficient. The stable resin matrix is not prone to biodegradation.

This polymerization mode can also improve the mechanical properties. Compared with light curing, high-temperature and high-pressure curing can prominently enhance the bending strength, Weibull modulus, hardness and density of resin [139,140]. At present, resin-based ceramic products cured at high temperature and high pressure have stronger mechanical properties [141]. This is because high temperature and high pressure can accelerate the molecular crosslinking reaction and shift the reaction equilibrium towards the positive direction [138,142,143]. Therefore, the resin composite can better resist chewing wear, and the monomer release rate is reduced.

#### 6.3.4. Reduction in Excess Water on the Bonding Interface

As mentioned above, the residual water in the dentin stops the adhesive from completely penetrating the demineralized collagen net, causing the separation of the hydrophilic part and hydrophobic part of the adhesive, forming cracks in the adhesive layer and increasing the invasion of bacteria. Excess water can aggravate biodegradation. Therefore, ethanol can be used instead of water, which not only ensures that the collagen net does not collapse, but also facilitates the penetration of adhesive into the collagen net to form a hybrid layer [105].

### 6.4. Improvement of the Oral Microenvironment

In addition to modifying the resin-based material itself to reduce the occurrence of biodegradation, recently, some new measures have been proposed to improve the oral microenvironment. Probiotic therapy is a novel method to prevent or treat caries, gingivitis or periodontitis [144]. The definition of probiotics is that “live microorganisms which, when administered in adequate amounts, confer a health benefit on the host” [145]. Most probiotics are Gram-positive bacteria, including the genera *Lactobacillus* or *Bifidobacterium* [146]. Reham et al. [147] cocultured the *Lactobacillus* supernatant with *S. mutans* suspension, finding that *Lactobacillus* sp. can inhibit tooth decay by impairing the growth and virulence properties of *S. mutans*. Therefore, when the concentration of *S. mutans* in the oral environment decreases, the resin-based materials are likely to be less susceptible to biodegradation. What is more, some unexplored variables can have a significant influence on the oral environment. The use of probiotics, postbiotics and natural compounds can modify clinical and microbiological parameters [148,149,150]. All these methods can be considered for clinical use. Table 1 summarizes the methods to reduce the occurrence of biodegradation.

## 7. Conclusions and Future Perspectives

Resin composites have the advantages of good plasticity and fluidity, strong adhesion, similar elastic modulus and color to dentin, and so on. However, there are also some problems with clinical application. Especially, the biodegradation factor is incredibly important and cannot be ignored. In fact, biodegradation can lead to the destruction of the bonding interface between resin and the tooth, resulting in secondary caries and prosthesis detachment. In this instance, biodegradation is derived chiefly from esterase from the saliva and bacteria, leading to the hydrolysis of ester bonds in resin and adhesive. It is also from endogenous and exogenous proteases. Under certain conditions, the dentin collagen net at the bonding interface is degraded by MMPs and cysteine cathepsin, resulting in cracks on the bonding interface. Biodegradation is accompanied by the production of Bis-HPPP, TEGMA, TEG, MA and other monomers. At the same time, these monomers can reversely promote the increase in the virulence of cariogenic bacteria and the accumulation of plaque, leading to more severe degradation.

Therefore, it is necessary to prevent or reduce biodegradation by some methods, involving adding some antibacterial components or enzyme inhibitors to resin-based materials or adhesives, or treating the exposed dentin bonding surface with crosslinking agents to improve the strength of collagen fibers and reduce the possibility of degradation by collagenase, etc.

However, as well as biodegradation, some other problems should also be focused on. Firstly, while using methods to reduce biodegradation, other factors affecting the adhesion of resin cannot be ignored, such as wetness isolation, light curing time and self-aging of resin [152,153]. Secondly, during the modification of resin, excessive fluorination can reduce the mechanical properties [114]. At the same time, due to chewing abrasion, antibacterial fillers will gradually be released, and the hardness of the resin will also be affected. Finally, excessive addition of antibiotics may induce drug-resistant strains [121].

Therefore, in order to solve the above problems, future research should focus on developing resins that can reduce biodegradation without weakening the mechanical properties. For example, replacing ester bonds with ether bonds can reinforce the stability of the polymer [115]. Moreover, more contact-killing composites can be designed to replace the traditional resin containing antibacterial fillers. Due to the fact that most bacteria cell membranes contain negative charges, the current researching direction is to add cationic compounds to monomers, which has a very broad application prospect [154]. In addition, application of mesoporous silica nanoparticles is also a new direction of current research; studies have shown that they can significantly prolong the time of drug release as drug carriers [118].

## Figures and Tables

**Table 1:** Summary of the methods to reduce the occurrence of biodegradation.

Methods	Mechanisms	Advantages	Drawbacks
Protection of ester bonds by fluorination	Add fluorinated groups to the hydrocarbon polymer substrate using chemical modification methods; replace Bis-GMA or TEGDMA with fluorinated monomers to reduce the release of unreacted monomers.	The hydrophobicity of fluorinated monomers can reduce the sensitivity of ester bonds to hydrolysis; the internal fluorocarbon chain of modified monomer is larger than that of hydrocarbons, providing steric hindrance to further protect ester bonds.	Reduce the mechanical properties (hardness, bending resistance, etc.) of modified resins.
Antibacterial fillers	Modified resin release antibacterial fillers to reduce bacteria attached to restorations, avoiding the hydrolysis of esterase.	High local doses of antimicrobial agents at a specific site without exceeding systemic toxicity; strong and broad-spectrum antimicrobial characteristics.	Have a long-term release rate and cannot maintain continuous antibacterial properties.
Antibacterial monomers	These monomers contain positive charges, which can attract negatively charged bacteria, thus changing their cell membrane permeability and destroying the cell membrane to achieve antibacterial effect.	More stable and not easy to induce drug-resistant strains.	Increase the water absorption of resin and lead to a reduction in mechanical strength.
Antibacterial adhesives	Reduce the accumulation of bacteria near the bonding interface, thereby reducing biodegradation.	Has excellent bonding strength, long-term antibacterial effect and the ability of promoting remineralization.	Continuous consumption of antibacterial ingredients.
Inhibition of protease hydrolysis of collagen	Inhibit MMP and cysteine cathepsin activity to improve the stability of the bonding interface.	Improve bonding strength and maintain interface durability.	Some MMP inhibitors (e.g., CHX) have a poor long-term effect.
Stabilization of dentin collagen net	Add crosslinking agent to stabilize the structure of collagen and surround collagen, making it more resistant to enzymatic degradation.	Natural crosslinking agent has the characteristics of low toxicity and sustainability. Some physical crosslinkers (riboflavin) can inhibit the activities of MMPs.	Chemical reagents have certain cytotoxicity.
High-temperature and high-pressure initiation conditions	The polymerization of resin monomers under high temperature and high pressure can significantly reduce the release of unreacted monomer.	Improve the mechanical properties [139,140].	The conversion of double bonds in monomers is not high with only high-pressure treatment, but high temperature can enhance the fluidity of monomers and improve the conversion of double bonds.
Reduction in excess water on the bonding interface	Use ethanol to replace water contained in the adhesives adhesive to remove free water and half of the third layer of bound water, which decreases the separation between the collagen matrix and resin monomers and, in turn, the possibility of activating collagenolytic enzymes.	Ethanol can ensure that the collagen net does not collapse and facilitates the penetration of adhesive into the collagen net to form a hybrid layer.	Have a lower inhibition effect on MMPs than alcohols with four methylene groups.
The use of probiotic therapy	Probiotics can inhibit the growth and virulence properties of *S. mutans*, thus decreasing the extent of biodegradation.	Probiotics protect against some oral diseases, while reducing biodegradation.	Some probiotics, such as *bifidobacterial*, are acidogenic and aciduric, and have been associated with both enamel and dentinal caries lesions [151].

## Data Availability

Not applicable.
